# Mapping Slow Mobility Users' Unhealthy Exposure to Air Pollution in London

**DOI:** 10.1002/hsr2.72005

**Published:** 2026-03-09

**Authors:** Yijing Li, Zhen Zhu, Sijie Tan, Rongsheng Zheng

**Affiliations:** ^1^ Department of Informatics King's College London London UK; ^2^ Kent Business School University of Kent Kent UK

**Keywords:** 15‐min city, air pollution, public health, slow mobility, urban planning

## Abstract

**Background and Aims:**

Slow mobility, characterized by modes of transportation such as walking and cycling, is often promoted for its health benefits and environmental friendliness. However, users of slow mobility face prolonged and direct exposure to their immediate environment, potentially increasing their susceptibility to air pollution compared to those using faster transportation modes. This study aims to quantify and spatially analyze the air pollution exposure associated with slow mobility in urban areas, using London as a case study, to inform healthier and more sustainable planning practices.

**Methods:**

This paper proposes a data‐driven framework to assess the relationship between slow mobility and air pollution exposure as a health risk, with a focus on London as a case study. The study overlays London's air pollution data of PM_2.5_, PM_10_, and NO_x_ onto the accessibility mapping based on the concept of “15‐min city” and point‐of‐interest data for London sourced from *OpenStreetMap*.

**Results:**

The findings indicate that Central London exhibits both high accessibility and high air pollution exposure. Moreover, NO_x_ levels are consistently higher than PM_10_ and PM_2.5_ for slow mobility users across London. However, projections for 2030 suggest significant improvements in air quality throughout the city.

**Conclusion:**

This paper provides empirical evidence supporting a comprehensive and people‐centered urban development philosophy, which requires extensive cooperation and joint efforts among stakeholders to achieve strategic goals for London by 2030.

## 1 Introduction

Urban planning has increasingly embraced a more human‐centered approach [1, 2, 3, 4], particularly in the post‐pandemic era, where urban lifestyles have undergone significant changes [5, 6, 7, 8]. As a result, there is a growing urgency to adopt more humane and sustainable urban planning methods to reshape our cities. A prominent model is the “15‐min city” [9], which advocates that essential services be accessible within a 15‐min walking time. This definition of slow mobility is adopted in this study.

However, many cities around the world are grappling with air pollution issues [10, 11, 12] that have clear detrimental effects on both physical and mental health [13, 14, 15, 16]. Slow mobility users are particularly vulnerable compared to those using faster modes of transportation, as they are exposed to harmful environments for longer periods and more directly [17, 18]. For instance, studies in Beijing, China [19], Jinan, China [20], New York, USA [21], Paris, France [22], and Sydney, Australia [23] have demonstrated that walkable areas do not necessarily align with areas of low pollution, creating trade‐offs between mobility, equity, and health.

While efforts have been made to quantify slow mobility [24] and related concepts such as walking accessibility [25, 26] and walkability [27, 28, 29, 30], there is a notable gap in research empirically examining the relationship between slow mobility and exposure to health risks such as air pollution. To address this gap, this study proposes an empirical framework to assess the relationship and map the exposure of slow mobility users to air pollution, focusing on London as a case study.

London serves as an ideal case study for examining slow mobility and air pollution for several reasons. Firstly, as a major global city with a diverse population and extensive public transport system, London presents a unique urban environment where walking and other forms of slow mobility are prevalent [31, 32]. This makes it an important context for studying the accessibility of services and the resulting exposure to air pollution. Secondly, London has been at the forefront of implementing policies aimed at improving air quality, such as the Ultra Low Emission Zone (ULEZ) and initiatives to promote walking and cycling [33, 34]. These policies create a dynamic setting to analyze the relationship between slow mobility practices and air pollution exposure, particularly as the city seeks to balance accessibility with environmental health. Last but not least, London offers relatively comprehensive data availability compared to other cities, including point‐of‐interest information and air quality measurements, which enables detailed analysis of slow mobility patterns and pollutant exposure.

## 2 Data and Methods

This study aims to map the exposure of slow mobility users to air pollution by overlaying air pollution data onto the accessibility mapping based on the concept of “15‐min city”. The “15‐min city” accessibility mapping serves as an indicator of areas where slow mobility is most likely prevalent. By analyzing the walking accessibility of various services and facilities, it assesses whether the current urban layout aligns with the concept of “15‐min city”. Moreover, superimposing air pollution data onto this map allows this study to assess the exposure of slow mobility users to air pollution and the related health risks. London is selected as a case study to explore how these interactions play out in a real‐world urban setting.

The research workflow is designed in three main steps, including data retrieval and preparation, calculation of 15‐min walking areas from origin residential locations to various land use destinations, and integration with air quality data to assess exposure levels.

Step 1: The research firstly retrieved point‐of‐interest data for London from *OpenStreetMap* by their land uses including residential areas as well as five types of services for accessibility measurements: core services for daily necessities, healthcare services, leisure services, public transport services and retail stores services. The research design rationale is to identify all nearest service points to target residential area, by calculating the walking time (at an average walking speed of 1.4 m/s) alongside computed pedestrian network map of London. Meanwhile, the ready‐to‐use air quality data were derived from London Data Store (Table 1) with modeled 2019, 2025, and 2030 ground level concentrations of annual mean NO_x_, PM_10_ and PM_2.5_ in µg/m^3^ (microgram per cubic meter) at 20 m grid resolution in GeoTiff format.

**Table 1:** Data sources.

	Name	Source	Notes
Air quality data	London Atmospheric Emissions Inventory (LAEI) 2019	https://data.london.gov.uk/dataset/london-atmospheric-emissions-inventory--laei--2019/	NO_x_, PM_10_, and PM_2.5_ emissions concentrations data for London in 2019, and predictions in 2025, and 2030
Slow mobility accessibility	Point‐of‐interest locations	https://wiki.openstreetmap.org/wiki/Key:amenity	Daily necessity locations geodata retrieved from Open Street Map API.

*Note:* London Local Air Quality Management (LLAQM), London Atmospheric Emissions Inventory (LAEI).

To prepare the locational data for residential areas and five types of services points, data transformation and cleaning were conducted, including ensuring consistent coordinate systems to align with the city's pedestrian network map. Moreover, centroids of polygonal geometric objects were computed to represent the corresponding service facilities' geolocations, as complex geometries with multi‐point shapes usually were calculated based on the points' locations to identify the closest road network nodes.

Step 2: The Python package *OSMnx* was deployed to retrieve the London street network from *OpenStreetMap* in geographically shapefile format, in preparation for constructing the pedestrian (walking) road network and calculating walking paths. Residential area recognized from Step 1 was used as Origin (O) area, and the walking time from each O to the nearest service point in each category (D) was calculated so to identify the nearest street network node. This process was repeated for each pair of O‐D until the walking times from all residential areas (O) to each category of service points (D) were calculated and recorded. The walking time T was calculated using Equation (1):

(1) $$T=\frac{D_{\mathrm{ij}}}{S}$$

where S is a constant value for the average adult's walking speed, at 1.4 m/s; $D_{\mathrm{ij}}$ is the distance between the origin node i (the i‐th residential neighborhood) and destination node j (the j‐th nearest service point) in the London street network, which allows for the identification of walking time alongside the shortest path in the street network. Due to computational capacity limits, the London street network and land use functioning data were first processed in subregions, then aggregated for city‐wide presentation.

**Figure 1:**
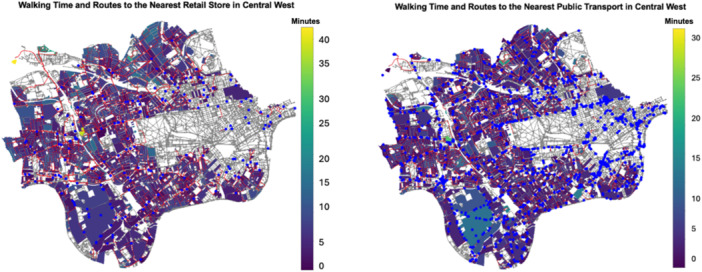
Central West London residents walking time to retail services (left) and to public transport services (right).

An example for the outputs in Central West London was illustrated in Figure 1, including two boroughs, City of Westminster and Kensington and Chelsea. The figure presents walking accessibility maps from residential areas to two selected services, retail services (the left panel) and public transport services (the right panel). In Figure 1, service venues (points‐of‐interest) are represented by blue dots. The color intensity of residential area polygons indicates walking time to the respective services, with darker colors ranging from dark blue‐green to purple representing areas within a 15‐min walk. Residential areas with red boundaries show high accessibility to both public transport and retail services within 15 min. Moreover, accessibility to public transport appears better than to retail services in this subregion.

Step 3: After identifying the shortest paths from each target residential area to the nearest service points in each category using Equation (1), only those accessible within a 15‐min walking time were kept as 15‐min accessible neighborhoods. These neighborhoods then served as base layers for overlaying London's air pollution data, including PM_2.5_, PM_10_, and NO_x_, using spatial operations, to map and compute the corresponding pollutant exposures for the years 2019 and 2030 (predicted) [35]. Special attention was given to pollutant levels, as referenced in the World Health Organization global air quality guidelines [36], shown in Table 2.

**Table 2:** WHO air quality guidelines and estimated reference levels.

PM_10_	1 day	45 μg/m^3^
	Calendar year	15 μg/m^3^
PM_2.5_	1 day	15 μg/m^3^
	Calendar year	5 μg/m^3^
NO_2_	1 h	200 μg/m^3^
	1 day	25 μg/m^3^
	Calendar year	10 μg/m^3^

*Note:* 99th percentile (3–4 exceedance days per year). Updated 2021 guideline.

## 3 Results

**Figure 2:**
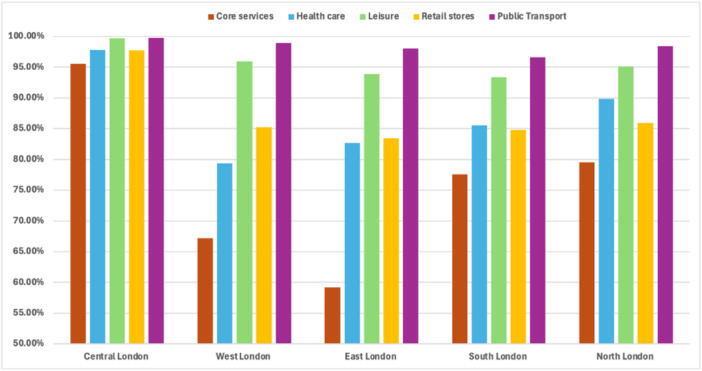
15‐min accessible neighborhoods by London regions.

Derived from *OpenStreetMap* data for London, 30,068 polygons were identified as residential areas, serving as the target areas for calculating 15‐min walking neighborhoods. The statistics in Figure 2 illustrate that the availability of 15‐min walking neighborhoods varies across different regions of London, with Central London ranking as the most accessible area (over 95% of all services can be reached on foot within 15 min).

Accessibility by walking also varies by service category. For instance, 98% of residents can access public transport services within 15 min, regardless of their location in London. Leisure services rank as the second most accessible category, whilst daily core services consistently rank the lowest, with about 82% of Londoners able to walk to them within 15 min, particularly in East London. Additionally, healthcare services are the least accessible in West London, with access rates falling below 80%, which is comparable to the accessibility of retail services.

With the 15‐min accessible residential areas identified, further analysis was conducted by overlaying these layers with data on air pollutant levels to generate statistics on exposure to each type of air pollutant encountered while walking within 15 min to access defined categories of services. The air pollutant layers used include PM_10_, PM_2.5_, and NO_x_ levels from 2019, reflecting the most recent air quality data, as well as parallel projected data for 2030 to indicate potential changes in relation to the air quality goals set for London. The projection modeling algorithm was accredited to Beevers et al. [37], with data released by Greater London Authority on their official data portal (https://data.london.gov.uk/dataset/london-atmospheric-emissions-inventory--laei--2019/).

**Figure 3:**
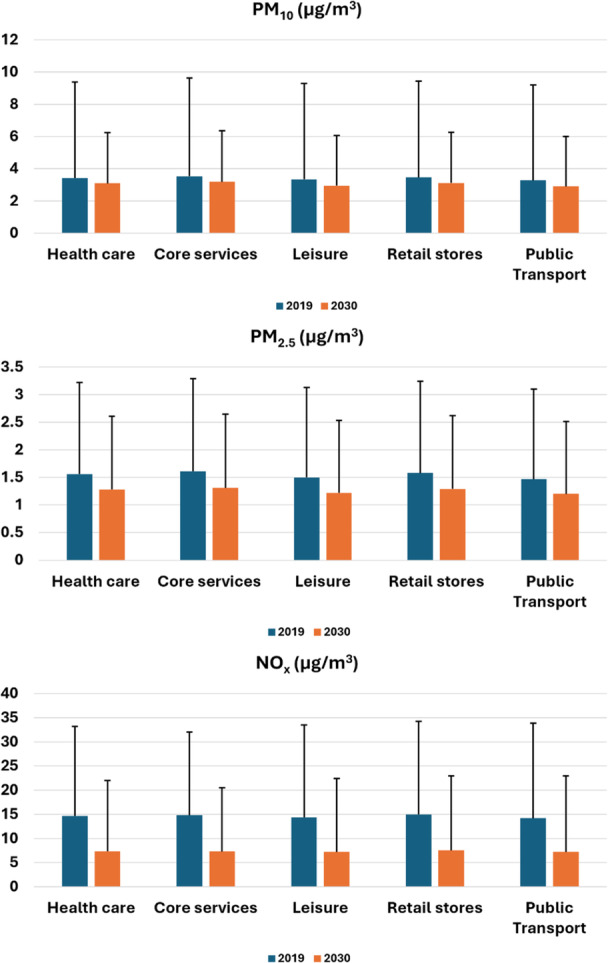
Air pollutants exposures for 15‐min accessible neighborhoods in 2019 and 2030.

Figure 3 shows the 15‐min accessible residential areas for each service category and their associated exposure to PM_10_, PM_2.5_, and NO_x_ respectively in 2019 and 2030 (predicted), with error bars representing one standard deviation. Slow mobility (walking) within 15 min is associated with lower exposures to pollutants when accessing leisure services and public transport services, but higher exposures when accessing daily essential core services and retail shopping. Furthermore, among residential areas accessing any services within a 15‐min walk, exposure levels to NO_x_ are generally higher than those for PM_10_ and PM_2.5_. This suggests the need for more detailed analysis in future research when finer‐grained data becomes available.

**Figure A1:**
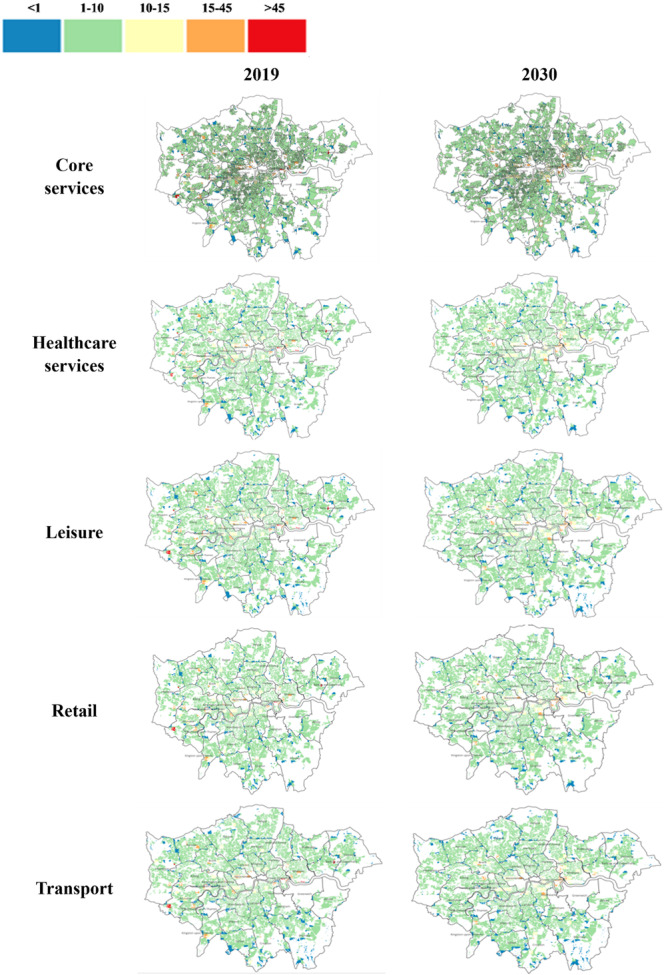
15‐min accessible neighborhoods exposure to PM_10_ (µg/m^3^).

**Figure A2:**
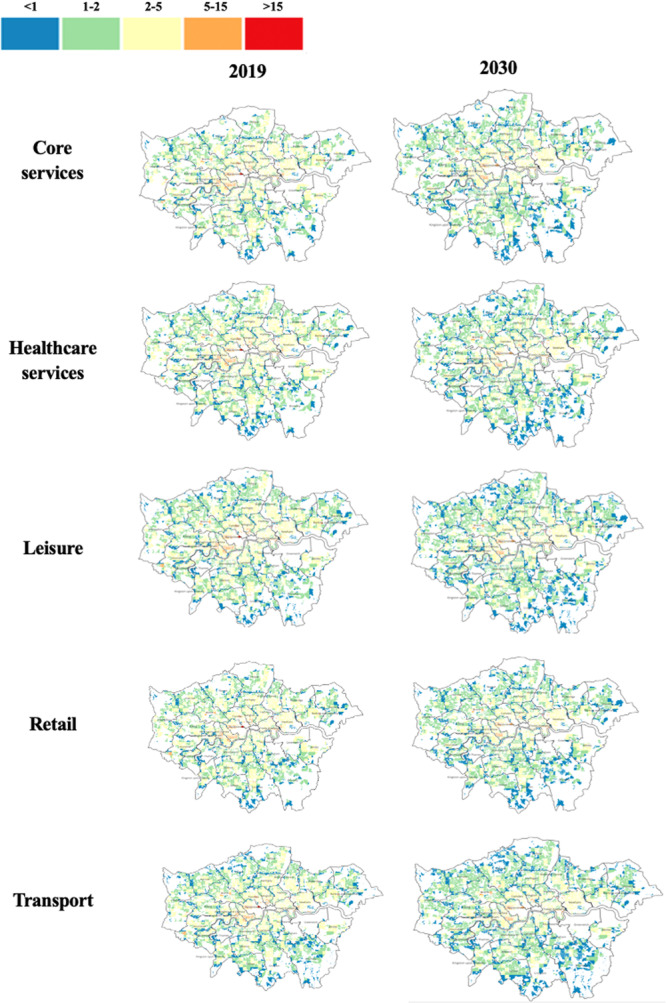
15‐min accessible neighborhoods exposure to PM_2.5_ (µg/m^3^).

**Figure A3:**
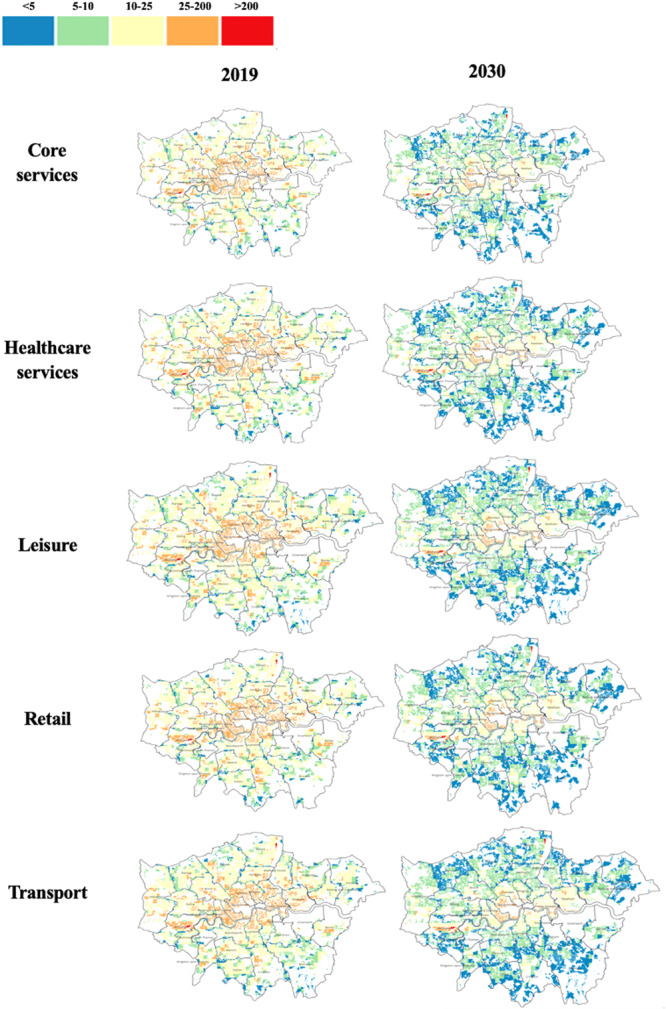
15‐min accessible neighborhoods exposure to NO_x_ (µg/m^3^).

To better understand air pollutant exposure during slow mobility to services, choropleth maps were drawn to illustrate Londoners' exposure to each type of pollutant (see Figures A1, A2, and A3 in the appendix for PM_10_, PM_2.5_, and NO_x_ respectively). Areas shown in blue and green indicate better air quality compared to those in yellow and orange, which exceed WHO guidelines. Red areas in Central London were identified as the most polluted. In 2019, PM_2.5_ (Figure A2) showed greater relative variability and a more uneven spatial distribution across London compared to PM_10_ (Figure A1), while NO_x_ (Figure A3) was identified as the most severe pollutant exposure.

Predictions for 2030 indicate a promising outlook for London's air quality, suggesting that slow mobility for accessing daily services within 15 min will become cleaner and healthier regarding air pollutant exposure. Significant improvements are expected for all three observed pollutants in most areas of London, particularly for NO_x_. However, Central London and locations such as Heathrow Airport are anticipated to continue experiencing relatively higher levels of air pollution (as indicated by orange and red colouring on the maps in the appendix).

## 4 Discussion

This study proposed an empirical framework to assess the relationship between slow mobility and exposure to air pollution as a health risk, using London as a case study. The point‐of‐interest data was retrieved from *OpenStreetMap* for identifying residential areas and five service categories, including core services for daily needs, healthcare, leisure, public transport, and retail. The shortest walking paths from each residential area to the nearest service in each category were calculated, and only those reachable within a 15‐min walk were classified as 15‐min accessible residential areas. These areas formed the base layers for measuring slow mobility use in London. The main finding is that accessibility varied by region and service type, with Central London being the most accessible region, while leisure and public transport were the most accessible services, and core services were the least accessible.

To explore the relationship between slow mobility and air pollution exposure, these base layers were overlaid with air pollution data for PM_2.5_, PM_10_, and NO_x_, representing exposure levels in 2019 and predicted levels for 2030. Central London exhibited both high accessibility and high air pollution exposure, reflecting the trade‐offs of agglomeration. NO_x_ levels were higher than PM_10_ and PM_2.5_ for slow mobility users. However, projections for 2030 indicate significant improvements in air quality across the city, offering a positive outlook for public health while maintaining accessibility [38].

This study contributes to ongoing discussions about the benefits and costs of agglomeration in urban environments [39, 40]. While previous studies often define accessibility in terms of faster modes of transportation such as highway access [39] and shorter travel times [40], this study applies the concept of “15‐min city”, focusing on accessibility to core services via slow modes of transportation. In relation to slow mobility, this study is closely aligned with the work of Milakis et al. [41]. However, whereas their study mainly discussed the negative environmental and social implications of producing slow mobility vehicles such as e‐scooters, this study emphasizes the health risks associated with air pollution exposure for slow mobility users.

Based on the concept of “15‐min city”, this study measured the use of slow mobility in London and provided empirical evidence supporting a comprehensive and people‐centered urban development philosophy, which requires extensive cooperation and joint efforts among stakeholders for successful implementation. When executed effectively, it can improve residents' well‐being, promote social equity and inclusiveness, and strengthen community cohesion. However, further actions and measures are needed to improve air quality in line with strategic goals for 2030.

Future studies should incorporate additional environmental and urban factors, such as green space availability, noise pollution, and heat stress, to build a more multidimensional understanding of the slow mobility experience. Including seasonal variations and mobility‐related pollution patterns can also help capture temporal dynamics often overlooked in static models. Furthermore, applying quantitative approaches such as exposure–response functions [16] could establish stronger links between pollutant levels and specific health outcomes for slow mobility users. These extensions would significantly enrich the evidence base and inform more comprehensive interventions toward London's strategic goals for 2030.

## 5 Conclusion

This study highlights a critical trade‐off in urban planning: areas with high accessibility for slow mobility users, such as Central London, often coincide with elevated levels of air pollution, particularly NO_x_. By integrating air quality data with the “15‐min city” framework, the analysis underscores the need for more nuanced planning that balances accessibility with environmental health. While future projections suggest overall air quality improvements by 2030, current patterns call for targeted interventions to protect slow mobility users in London.

## Author Contributions

**Yijing Li:** conceptualization, supervision, data curation, software, methodology, funding acquisition, writing – original draft, writing – review and editing, investigation, formal analysis, validation, and visualization. **Zhen Zhu:** conceptualization, formal analysis, writing – original draft, writing – review and editing, investigation, and validation. **Sijie Tan:** software, data curation, methodology, formal analysis, visualization, investigation, and validation. **Rongsheng Zheng:** software, data curation, methodology, formal analysis, visualization, investigation, and validation.

## Conflicts of Interest

The authors declare no conflicts of interest.

## Transparency Statement

The corresponding author, Zhen Zhu, affirms that this manuscript is an honest, accurate, and transparent account of the study being reported; that no important aspects of the study have been omitted; and that any discrepancies from the study as planned (and, if relevant, registered) have been explained.

## Data Availability

The data that support the findings of this study are available from the corresponding author upon reasonable request.

## 1

See Figure A1

See Figure A2

See Figure A3.
